# Virulence and Antibiotic Resistance Profiles of *Staphylococcus aureus* Isolated from Epidermal Growth Factor Receptor Inhibitors-Associated Skin Lesions

**DOI:** 10.3390/ijms26146595

**Published:** 2025-07-09

**Authors:** Mara-Mădălina Mihai, Iuliana Anghelescu, Alina Maria Holban, Irina Gheorghe-Barbu, Mariana-Carmen Chifiriuc, Lia-Mara Dițu, Cornelia-Ioana Ilie, Dan Anghelescu, Beatrice Bălăceanu-Gurău

**Affiliations:** 1 Department of Oncologic Dermatology, “Elias” Emergency University Hospital, “Carol Davila” University of Medicine and Pharmacy, 020021 Bucharest, Romania; mara.mihai@umfcd.ro (M.-M.M.); beatrice.balaceanu@drd.umfcd.ro (B.B.-G.); 2 Research Institute of the University of Bucharest, University of Bucharest, 050663 Bucharest, Romania; irina.gheorghe@bio.unibuc.ro (I.G.-B.); carmen.chifiriuc@unibuc.ro (M.-C.C.); lia_mara_d@yahoo.com (L.-M.D.); 3 Department of Histology, Titu Maiorescu University of Medicine and Pharmacy, 040441 Bucharest, Romania; iulia0lupu@gmail.com; 4 Department of Botany-Microbiology, Faculty of Biology, University of Bucharest, 030018 Bucharest, Romania; 5 Department of Microbiology-Immunology, Faculty of Biology, University of Bucharest, 050095 Bucharest, Romania; 6 Biological Sciences Division, Romanian Academy, 010071, Bucharest, Romania; 7 Department of Science and Engineering of Oxide Materials and Nanomaterials, Faculty of Applied Chemistry and Materials Science, National University of Science and Technology POLITEHNICA Bucharest, 011061 Bucharest, Romania; cornelia_ioana.ilie@upb.ro; 8 National Centre for Micro and Nanomaterials and National Centre for Food Safety, National University of Science and Technology POLITEHNICA Bucharest, 060042 Bucharest, Romania; 9 Department of Orthopedics and Traumatology, “Titu Maiorescu” University, 040441 Bucharest, Romania; dr.anghelescu@gmail.com; 10 Clinical Department 1 of Orthopedics and Traumatology, Colentina Hospital, 020125 Bucharest, Romania

**Keywords:** EGFR inhibitors, papulopustular eruptions, paronychia, *Staphylococcus aureus*, biofilm formation, antibiotic resistance, genotypic profiling, phenotypic resistance

## Abstract

Cutaneous adverse reactions (CARs) are common complications of epidermal growth factor receptor (EGFR) inhibitor therapy, with papulopustular eruptions and paronychia being the most frequent. Growing scientific evidence implies that *Staphylococcus aureus* is involved in the pathogenesis of these reactions. This observational prospective study characterized 42 *S. aureus* strains isolated from CARs, analyzing antibiotic resistance, biofilm formation, soluble virulence factors, and virulence/resistance genes using multiplex polymerase chain reaction (PCR). *S. aureus* was identified in 90% of lesions; in 33% of cases, nasal and skin isolates were genetically identical. High resistance rates were noted for penicillins (85%) and tetracyclines (57%), while all strains remained susceptible to fluoroquinolones, vancomycin, and rifampicin. All isolates formed biofilms, and DNase/esculinase production significantly correlated with CAR severity. An enzymatic score based on these markers was associated with an 18-fold increased risk of severe reactions. Genotypically, *clfA* and *clfB* were prevalent (85.7%), while exotoxin genes were less common. These findings support a key role for *S. aureus* in exacerbating CARs via antibiotic resistance, biofilm production, and the expression of virulence factor. Additionally, we emphasize the role of routine microbial screening—including nasal swabs—and therapy guided by antibiograms. Furthermore, the enzymatic score may further be validated as a predictive biomarker.

## 1. Introduction

Cutaneous adverse reactions (CARs) are the most prevalent side effects associated with the administration of Epidermal Growth Factor Receptor (EGFR) inhibitors, due to the pronounced expression of EGFR in the skin and its adnexal structures [1]. Among these dermatological lesions, the most frequently encountered are encapsulated within the PRIDE syndrome—an acronym representing Papulopustules, Paronychia, Regulatory abnormalities of hair growth, Itching, and Dryness [2]. The initiation and intensity of papulopustular eruptions have been demonstrated to positively correlate with both therapeutic response and overall survival [3,4]. Consequently, these eruptions are considered indicators of therapeutic efficacy and prognostic markers of clinical outcomes [3,5]. Scientific evidence suggests that dose escalation in patients exhibiting grade ≤1 toxicity may enhance response rates [6]. Moreover, patients who experience a variety of CARs have superior survival outcomes [7].

Papulopustular eruptions—commonly known as acneiform reactions—represent the predominant CARs, occurring with greater frequency in individuals receiving monoclonal antibodies such as cetuximab (80%) compared to those undergoing treatment with tyrosine kinase inhibitors like erlotinib (67%) [8,9]. Clinically, these lesions present as erythematous papules and follicular pustules devoid of comedones or cysts, primarily affecting seborrheic areas, and are frequently exacerbated in a cyclical manner after cetuximab infusions [10]. While such eruptions have prognostic relevance, they may also considerably diminish quality of life, often demanding dose adjustments in the oncological treatment regimens [3,4,5].

EGFR inhibition compromises epidermal barrier integrity by downregulating critical structural and antimicrobial constituents such as claudin-1, LL37, and RNase 7, thereby fostering colonization by *Staphylococcus aureus* and potentially worsening dermatological lesions through toxin-mediated pathways [11,12,13,14,15,16]. *S. aureus* represents a prevalent pathogen in nosocomial infections and poses a significant threat to immunocompromised individuals [17]. Its pathogenesis involves a complex interaction between host defense mechanisms and bacterial virulence determinants [18]. These factors encompass cell wall-associated adhesins—such as protein A, clumping factors A and B, and proteins that bind to fibronectin, collagen, elastin, and fibrinogen—alongside soluble proteins and exotoxins, including hemolysins, coagulases, enterotoxins, exfoliative toxins, toxic shock syndrome toxin-1, and Panton–Valentine leukocidin [19]. Adhesins and coagulases facilitate adherence to host cells and implanted devices, initiating a colonization process that may evolve into an infection [19,20].

The pathogenicity of *S. aureus* is driven not only by antibiotic resistance and production of a diverse array of virulence factors, but also by its capacity to form biofilms [21,22]. Biofilms are highly organized and dynamic microbial aggregates encapsulated within a self-generated extracellular mucopolysaccharide matrix, which exhibits compositional variability across different colonies [21,22,23]. This matrix, consisting of polysaccharides, lipids, nucleic acids, and proteins—derived from host or bacterial cell lysis or directly secreted—affords protection against antimicrobial agents and host immune responses [23,24,25,26]. It also hampers phagocytosis and attenuates local immune activity [27,28]. Furthermore, the close spatial arrangement within biofilms facilitates horizontal gene transfer, including the spread of antibiotic resistance genes [29]. The formation of biofilms has been intimately associated with chronic infections, reduced therapeutic efficacy, and heightened resistance, to the degree that biofilms have been likened to “primitive multicellular organisms” [30,31,32].

Given these patterns and the suspected role of microbial colonization—particularly with *S. aureus*—in the development and persistence of CARs, we conducted an observational, prospective investigation in the Oncology Departments of the Bucharest University Emergency Hospital, Elias University Emergency Hospital, and the Bucharest Oncology Institute, between May 2014 and January 2015, analyzing the phenotypic and genotypic characteristics of *S. aureus* strains isolated from papulopustular lesions and paronychia in patients undergoing EGFR inhibitor therapy. This study aimed to explore the link between bacterial virulence, antibiotic resistance, and the clinical severity of dermatologic toxicity. In Romania, data regarding bacterial colonization, antibiotic resistance profiles, or virulence traits in CARs is lacking. This is among the few prospective studies characterizing phenotypic and genotypic features of isolated strains and their resistance to antibiotics.

## 2. Results

### 2.1. Phenotypic and Genotypic Characterization of S. aureus Strains Isolated from Papulopustular Lesions and Paronychia Arising During EGFR Inhibitor Therapy

Multiple bacterial species were isolated from papulopustular lesions and paronychia: *S. aureus* (42 strains), *Serratia marcescens* (5 strains), *Pseudomonas aeruginosa* (4 strains), *Enterococcus faecalis* (2 strains), *Klebsiella oxytoca* (2 strains), *Acinetobacter lwoffii* (2 strains), *Citrobacter diversus* (1 strain), and *Staphylococcus epidermidis* (1 strain).

*S. aureus* strains isolated from papulopustular reactions (36 strains) and paronychia (6 strains) were tested to evaluate their ability to form biofilms and express soluble virulence factors. Additionally, multiplex PCR tests were performed to identify eight bacterial genes encoding extracellular or cell wall-associated virulence factors, as well as 15 genes associated with methicillin resistance—and consequently resistance to β-lactam antibiotics.

#### 2.1.1. Biofilm Formation Capacity

**Figure 1:**
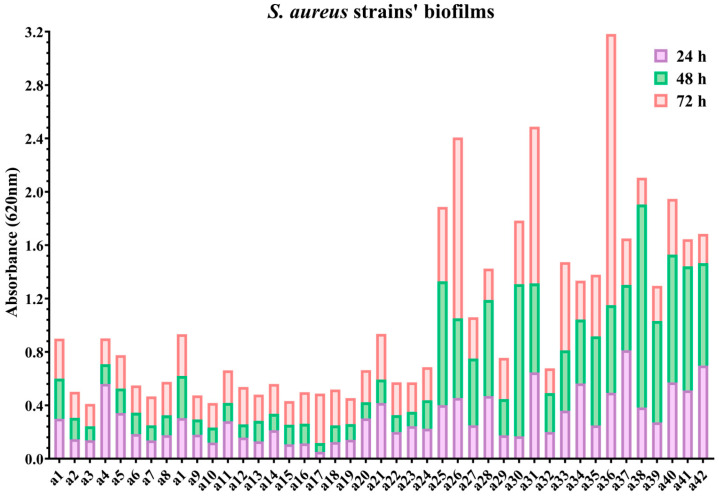
Biofilm formation of 42 *S. aureus* strains over 24, 48, and 72 h, measured by absorbance at 620 nm. All isolates demonstrate biofilm production, with increasing intensity over time. Strains a 25, a26, a30, a31, a36, a38 and a40 exhibited the strongest biofilm formation, while others showed moderate to low levels. Overall, biofilm-forming capacity is variable and strain-dependent.

All *S. aureus* strains demonstrated biofilm formation, with variable intensity depending on the lesion source, the strains isolated from paronychia exhibiting greater biofilm-forming capacity compared to those isolated from acneiform reactions (Figure 1).

We calculated Spearman correlation coefficients (ρ) between the severity of adverse reactions, as assessed by the CTCAE v4.03 score, and the biofilm-forming capacity at 24, 48, and 72 h of *S. aureus* strains isolated from certain lesions (Table 1). No statistically significant correlations between the biofilm-forming capacity of *S. aureus* strains and the severity of adverse reactions have been found.

**Figure 2:**
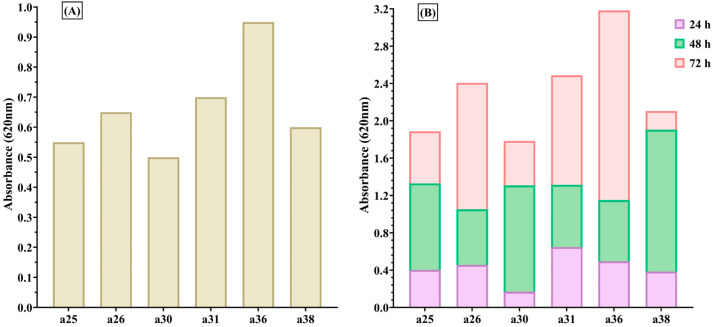
Biofilm-forming capacity of *S. aureus* strains isolated from paronychia; (**A**) mean absorbance at 72 h shows enhanced biofilm formation in strains a25, a26, a30, a31, a36, and a38; (**B**) time-course analysis (24 h—purple, 48 h—green, 72 h—red) demonstrates consistent biofilm development, with the highest levels observed at 72 h.

Strains isolated from paronychia exhibited greater biofilm-forming capacity compared to those isolated from acneiform reactions (Figure 2). The mean absorbance (OD) values at 24, 48, and 72 h for *S. aureus* strains from paronychia ranged between 0.594667 and 1.061, with an average of 0.769666 (Figure 2).

#### 2.1.2. Expression of Soluble Virulence Factors

**Figure 3:**
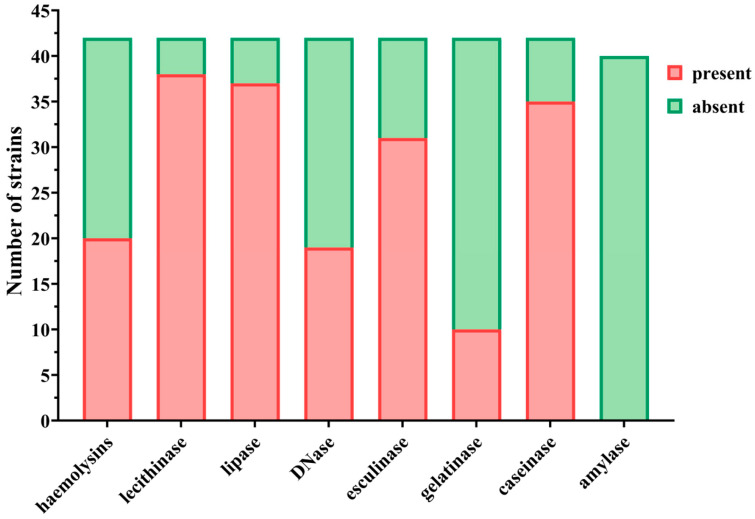
Expression of soluble virulence factors in *S. aureus* strains. Pore-forming toxins (haemolysins, lecithinase, lipase) were frequently expressed, indicating strong potential for tissue invasion. Among exotoxins, esculinase and caseinase were most prevalent, suggesting roles in iron acquisition and extracellular matrix degradation. Gelatinase and DNase were less commonly detected, while amylase was absent in all strains.

**Figure 4:**
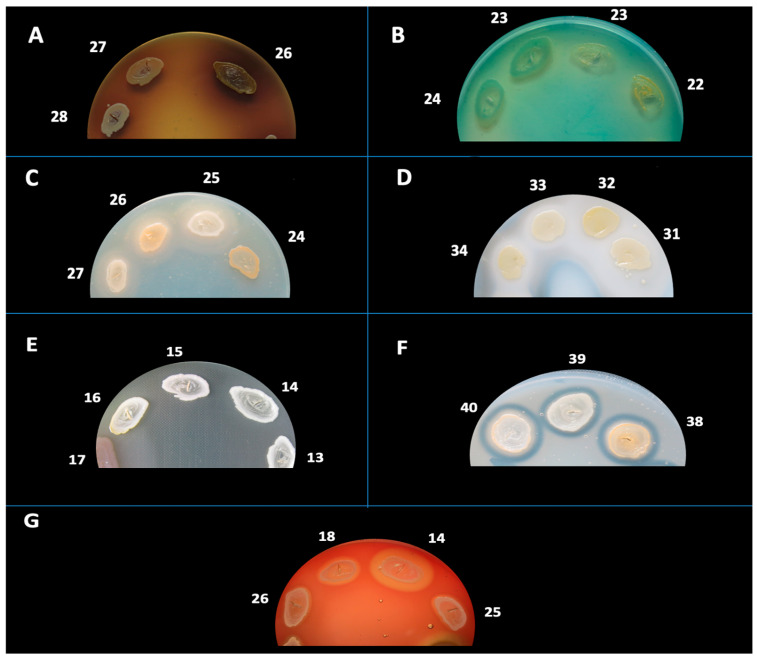
Phenotypic detection of soluble virulence factors produced by *S. aureus* isolates. Representative images of enzymatic activity are shown for: (**A**) esculinase (esculin hydrolysis on bile-esculin agar), (**B**) DNase (DNA hydrolysis with methyl green), (**C**) lipase (lipid degradation on spirit blue agar), (**D**) caseinase (casein hydrolysis), (**E**) gelatinase (gelatin liquefaction), (**F**) lecithinase (lecithin degradation on egg-yolk agar), and (**G**) hemolysins (hemolysis on blood agar). White numeric labels indicate the strain identifiers corresponding to individual *S. aureus* isolates. Numbers refer to strain IDs. Petri dish diameter = 90 mm. Scale bar = 10 mm.

The phenotypic expression of soluble virulence factors for the analyzed *S. aureus* strains was investigated through cultivation on media supplemented with enzyme-specific substrates (Figure 3 and Figure 4). These virulence determinants were classified into two major groups: exotoxins (DNase, esculinase, gelatinase, caseinase, and amylase) and pore-forming toxins (alpha- and beta-hemolysins, lecithinase, and lipase).

**Figure 5:**
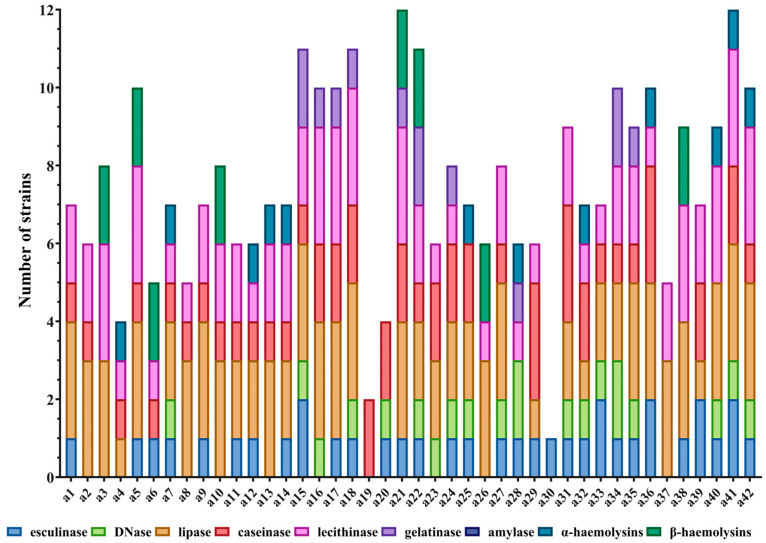
Distribution of soluble virulence factors in *S. aureus* strains. The stacked bar chart displays the number and variety of enzymatic virulence factors expressed by each of the 42 *S. aureus* isolates. Most strains produced multiple enzymes, including combinations of exotoxins (e.g., DNase, esculinase, gelatinase, caseinase, amylase) and pore-forming toxins (e.g., lecithinase, lipase, α- and β-haemolysins). Strains such as a15, a18, a21, a22, a36 and a41 demonstrated the highest cumulative enzymatic activity, reflecting a potentially greater pathogenic capacity.

The analyzed *S. aureus* isolates exhibited a pronounced potential for local tissue invasion and systemic dissemination (Figure 3 and Figure 5). This was attributed to the production of pore-forming enzymes, as well as esculinase—which facilitates iron acquisition necessary for bacterial metabolism, and caseinase—a proteolytic enzyme implicated in the degradation of the extracellular matrix of connective tissues.

Lecithinase was the most frequently expressed soluble virulence factor among the *S. aureus* isolates, being identified for 38 strains (90.47%), followed by lipase for 37 strains (88.09%), caseinase for 35 strains (83.33%), and esculinase for 31 strains (73.80%) (Figure 5). Hemolysins were expressed by 20 strains (47.61%), DNase by 19 strains (45.23%), and gelatinase by only 10 strains (23.80%) (Figure 3). Amylase expression was absent in all isolates (Figure 5).

**Figure 6:**
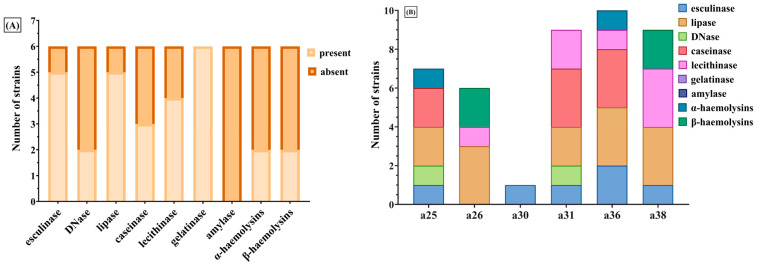
Soluble virulence factor profiles in *S. aureus* strains isolated from paronychia; (**A**) overall presence or absence of nine enzymatic virulence factors among six selected strains; (**B**) stacked bar representation of individual virulence profiles for strains a25, a26, a30, a31, a36, and a38. Most strains exhibited multiple enzymatic activities, with a36 and a38 expressing the broadest range of virulence factors. These profiles reflect a heightened pathogenic potential among *S. aureus* isolates from paronychia.

Focusing specifically on strains isolated from paronychia lesions, the majority (83.33%) exhibited the ability to produce lipase and esculinase (Figure 6). In contrast, only two-thirds of these strains expressed lecithinase and hemolysins, while caseinase was detected in 50% of the isolates (Figure 6). DNase was the least frequently expressed virulence factor in this subset (33.33%), and neither amylase nor gelatinase were detected in any of the *S. aureus* strains isolated from paronychia (Figure 6).

#### 2.1.3. Correlation Between Virulence Factors and Clinical Severity

The virulence potential of each *S. aureus* strain was quantified using a cumulative score based on the production level of individual virulence factors, rated semiquantitatively from 0 to 3. A composite virulence index for each *S. aureus* strain was calculated by summing the individual scores for all expressed virulence factors. Hemolytic activity was scored as γ = 0, α = 1, and β = 2. Amylase was not expressed by any strain and was thus excluded from the scoring.

Spearman’s rank correlation coefficient (ρ) was used to assess the relationship between the expression values of each virulence factor and the severity of the cutaneous adverse reaction at the start of treatment, as measured by the CTCAE severity score (Table 2).

A statistically significant positive correlation was found between DNase expression and the severity of cutaneous adverse reactions, as measured by the CTCAE 4.0 scale (ρ = 0.30, *p* = 0.047) (Table 2). Other individual factors such as lipase, lecithinase, and hemolysins did not demonstrate significant associations with severity (Table 2).

We investigated the potential correlation between the virulence score and the CTCAE severity of the primary cutaneous adverse reaction from which the *S. aureus* strain was isolated at treatment initiation, using Spearman’s rank correlation coefficient (ρ). A total virulence score, representing the sum of all expressed virulence markers per strain, showed a modest but statistically significant correlation with reaction severity (ρ = 0.34, *p* = 0.025).

The previously calculated total score assumed that all enzymes contributed equally to the correlation with CTCAE severity. To refine the analysis, a principal component analysis (PCA) was performed (Table 3), but no additional associations between the first four components and CTCAE severity were identified.

The previous plots indicate that no significant correlations exist between the principal components and the severity of the CTCAE-rated adverse reactions, suggesting that the only potential association between enzyme production level and clinical severity involves DNase and esculinase. Consequently, we calculated a simplified enzymatic score based solely on DNase and esculinase expression that also showed a significant correlation with severity, which was then correlated with CTCAE severity.

The enzymatic score ranged from 0 to 3, with distribution as follows: score 0 (21.4%), score 1 (33.3%), score 2 (33.3%), and score 3 (11.9%) (Table 4).

We further investigated whether there is an association between the CTCAE severity score and the enzymatic score using ordinal logistic regression, where the dependent variable (“outcome”) was the CTCAE severity and the independent variable (“predictor”) was the enzymatic score (Table 5).

The ordinal logistic regression revealed that strains with an enzymatic score of 3 were associated with an 18-fold increased risk of severe cutaneous reactions compared to strains with a score of 0 (Table 5). However, this association should be interpreted cautiously given the limited number of high-scoring strains.

#### 2.1.4. Genotypic Characterization of Virulence Factors

PCR-based genotypic profiling revealed that 38 out of the 42 *S. aureus* strains carried at least one of eight virulence-associated genes, either encoding extracellular toxins or cell wall-associated proteins, including *hlg* (γ-hemolysin), *lpv* (Panton–Valentine leukocidin), *fib* (fibrinogen-binding protein), *clfA* and *clfB* (clumping factors A and B), *bbp* (bone sialoprotein-binding protein), *fnbB* (fibronectin-binding protein B), and *ebps* (elastin-binding protein).

The most frequently detected were *clfA* and *clfB*, identified in 36 strains (85.71%) (Table 6). The *clfA* and *clfB* genes encode clumping factors involved in adherence to fibrinogen-coated surfaces and agglutination with plasma proteins. The *fib* gene, which encodes a fibrinogen-binding protein, was identified in 15 strains (35.71%) (Table 6). Only seven strains (16.66%) expressed the *hlg* gene encoding γ-hemolysin (Table 6). None of the isolates expressed the *ebps, fnbB*, or *lpv* genes.

**Figure 7:**
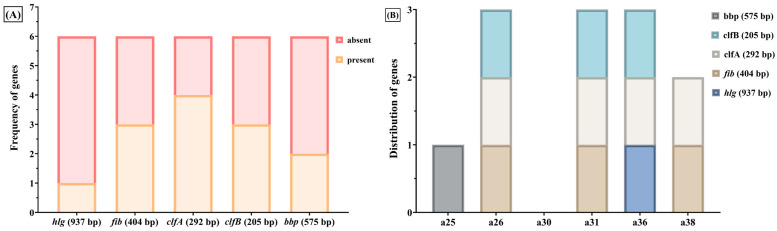
Adhesion gene profiles in *S. aureus* strains. (**A**) Frequency of five adhesion-related genes (*clfA, clfB, fib, bbp, hlg*) across the analyzed *S. aureus* isolates; (**B**) distribution of adhesion genes among *S. aureus* strains isolated from paronychia (a25, a26, a30, a31, a36, a38). The *fib* gene was expressed in 50% of these strains, while *bbp* was detected in one third. None of these isolates expressed the *hlg* gene. All strains from acneiform eruptions expressed *clfA* and *clfB*, while *bbp* was absent.

Among the *S. aureus* strains isolated from paronychia lesions, half of them expressed the *fib* gene encoding the fibrinogen-binding protein, and one-third expressed the *bbp* gene (Figure 7). Notably, all *S. aureus* strains isolated from acneiform eruptions expressed the *clfA* and *clfB* genes, while none of them expressed the *bbp* gene (Figure 7).

**Figure 8:**
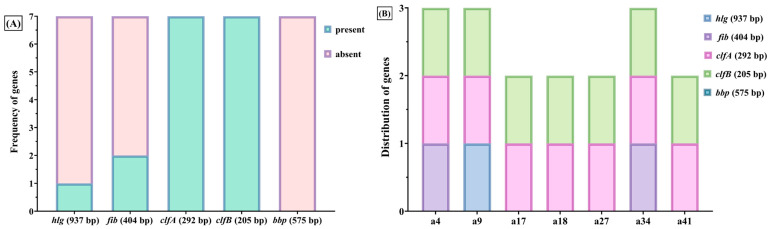
(**A**) Presence or absence of five adhesion genes (*hlg*, *fib*, *clfA*, *clfB*, *bbp*) in strains isolated from acneiform lesions. The y-axis represents the number of strains in which each gene was detected (present) or not detected (absent). (**B**) Stacked representation showing gene distribution in selected acneiform isolates (a4, a9, a17, a18, a27, a34, a41). Each colored segment corresponds to a detected gene, with a maximum of five genes per strain. The y-axis indicates the number of adhesion-related genes expressed per strain. All strains expressed *clfA* and *clfB*, while *bbp* was entirely absent. This uniform profile contrasts with the more heterogeneous gene patterns observed in strains from paronychia.

Strikingly, all isolates from acneiform lesions expressed both *clfA* and *clfB*, but none expressed *bbp*, suggesting a specific adhesion profile associated with this clinical pattern (Figure 8). Conversely, paronychia isolates exhibited higher heterogeneity, with expression of both *fib* and *bbp* observed in a subset of cases (Figure 8).

### 2.2. Phenotypic and Genotypic Characterization of Antibiotic Resistance in S. aureus Strains Isolated from Papulopustular Lesions and Paronychia Associated with Epidermal Growth Factor Receptor Inhibitor Therapy

#### 2.2.1. Phenotypic Resistance to Antibiotics

The *S. aureus* strains studied exhibited a high degree of resistance to antibiotics from various classes (Table 7, Supplementary Table S1). Phenotypic antibiotic susceptibility testing of *S. aureus* strains isolated from cutaneous lesions in patients receiving EGFR inhibitor therapy revealed a high degree of resistance to penicillins. Specifically, 83.33% of isolates were resistant to penicillin, and 16.665% to methicillin. Regarding macrolides, 45.23% of isolates were resistant to erythromycin, and 42.85% to clindamycin. All tested strains retained full sensitivity to linezolid, vancomycin, gentamicin, rifampicin, tobramycin, chloramphenicol, fusidic acid, and fluoroquinolones (ciprofloxacin, ofloxacin).

However, resistance to tetracyclines—frequently used in both prophylactic and therapeutic management of EGFR inhibitor-induced reactions—was notably high, with 57.14% of isolates resistant to tetracycline and doxycycline (Table 8). These findings underscore the importance of susceptibility testing prior to initiating tetracycline-based regimens in this patient population.

The CTCAE severity scores associated with the seven methicillin-resistant (MRSA) isolates ranged from grade 1 to grade 3. These are detailed in Table 8.

#### 2.2.2. Genotypic Resistance Profiles

The presence of 15 genes associated with methicillin resistance—and consequently resistance to β-lactam antibiotics—was investigated using PCR-based assays.

Genotypic analysis of the 42 *S. aureus* isolates revealed a low prevalence of key resistance genes: *mecA* in three strains (7.14%), *mecI* in four strains (9.52%), and *ccrB2* in three strains (7.14%) (Table 9).

SCC*mec* type IV gene was identified more frequently, being detected in nine isolates (21.42%) (Table 9).

In contrast, SCC*mec* type I and SCC*mec* III J1 were found in only two isolates (4.76%). The *kdp* gene was expressed in a single strain (Table 9).

SCC*mec* cassette types III, V, and II were not detected in any of the *S. aureus* isolates analyzed.

## 3. Discussion

The papulopustular eruption was reported in most patients, of whom the majority were undergoing treatment with cetuximab and only a few with erlotinib. Severe paronychia manifested in six patients receiving high-dose cetuximab, generally in conjunction with FOLFOX or FOLFIRI regimens. These lesions emerged after an eight-week treatment period as painful inflammation of the periungual folds, frequently exacerbated by the formation of abscesses or pyogenic granulomas. Although dose reductions were rare, Grade 2 papulopustular eruptions and paronychia significantly affected quality of life in 38% of the cohort.

Initially, the administration of systemic antibiotics was restricted to the period following the onset of CARs; however, emerging evidence supporting the prophylactic use of tetracyclines (200 mg/day), in conjunction with emollients and appropriate skin care, has illustrated a greater than 50% reduction in Grade 2 or lower CARs, thereby supporting their integration into standardized care protocols [9,33,34]. Despite a rising incidence of tetracycline-resistant *S. aureus* strains, doxycycline continues to exhibit clinical relevance due to its anti-inflammatory properties, which include the inhibition of matrix metalloproteinases [35,36]. CARs typically intensify during the initial month of treatment and subsequently achieve stabilization and improvement by the 6–8 week mark, underscoring the critical need for early prophylactic interventions [37]. Although CAR severity has functioned as a pharmacodynamic indicator in clinical trials, the extensive implementation of prophylactic measures may potentially diminish its prognostic significance [6]. In the context of clinical practice, dosing modifications for cetuximab are not predicated upon the grading of CARs [6].

Microbiological evaluations revealed the presence of *S. aureus* in 90% of patients exhibiting CARs. Additional pathogenic organisms included *S. marcescens, C. diversus K. oxytoca, P. aeruginosa, S. epidermidis,* and *E. faecalis*. Importantly, no instances of systemic infections were reported. Sampling methodologies were designed to ensure that the isolated pathogens accurately represented true infections rather than mere surface colonization [38,39]. Moreover, approximately one-third of patients included in the study carried nasal strains that were genetically and phenotypically congruent with those isolated from skin lesions, implying autoinoculation as a plausible mechanism. This finding underscores the significance of nasal colonization not only as a reservoir for *S. aureus* but also as a modifiable risk factor that may contribute to the persistence or relapse of dermatologic toxicity. The high concordance between nasal and cutaneous isolates highlights the need for targeted screening and decolonization strategies—particularly in patients with recurrent or severe CARs—to improve therapeutic outcomes and reduce lesion chronicity.

Antibiotic susceptibility evaluations indicated elevated resistance rates among *S. aureus* isolates, particularly concerning penicillins (85%) and tetracyclines (57%). All strains maintained susceptibility to fluoroquinolones, linezolid, vancomycin, and rifampicin. Genotypic analyses revealed the presence of SCC*mec* type IV in roughly 20% of strains, suggesting a potential community-acquired origin [40,41]. The *mecA* gene was identified in only 7% of isolates, which may be attributable to alternative resistance mechanisms or genetic loss during subculturing procedures [42,43,44].

All isolates demonstrated the capacity for biofilm formation, with strains originating from paronychia and late-onset papulopustular eruptions exhibiting more pronounced biofilm production. This observation suggests a potential correlation with chronicity and resistance to treatment [45,46]. Phenotypically, all strains displayed multiple virulence factors, with high prevalence rates of hemolysins, lecithinase, lipase, esculinase, and caseinase. Conversely, gelatinase was infrequently detected (23.8%), while DNase activity was recorded in 45.2% of strains.

Virulence index scores, derived from enzyme expression profiles, exhibited a weak yet statistically significant correlation with CTCAE-based grading of CARs severity. Among the evaluated factors, DNase and esculinase emerged as the most predictive, with an enzymatic score of 3 conferring an 18-fold elevated risk for the development of severe CARs, making it a potentially robust marker for early identification of high-risk patients. Principal component analysis did not reveal stronger associations beyond these individual enzymes. The association between the enzymatic score and the clinical severity of cutaneous adverse reactions suggests that virulence factor profiling could serve as a predictive tool in clinical practice. Given the ease of phenotypic assessment through standard microbiological techniques, incorporating such enzymatic profiling into diagnostic workflows could allow for timely, risk-adapted therapeutic strategies. However, prospective validation in larger cohorts is necessary to determine its reproducibility and clinical utility. Although we characterized both phenotypic and genotypic virulence features, we did not directly correlate gene presence with enzymatic activity. This is a limitation of the current study and should be addressed in future investigations using larger datasets and transcriptomic validation to determine whether gene expression consistently translates into functional protein activity.

Genotypic profiling indicated a high expression of adhesion-related genes such as *clfA* and *clfB* (85.7%) and *fib* (~33%), while extracellular virulence genes, including *hlg* and *bbp*, were rarely identified. Notably, *ebps, fnbB,* and *lpv* were absent across all isolates. The dominance of cell wall-associated virulence genes may play a significant role in prolonging colonization and contributing to resistance against therapeutic interventions.

Protein A and the fibronectin-binding proteins A and B (encoded by *fnbA/fnbB*) facilitate the adherence to fibronectin in fibrin clots or extracellular matrices of the host and have been associated with the pathogenesis of endocarditis [19]. Clumping factors A and B (*clfA, clfB*) promote the binding of fibrinogen—where *clfA* specifically interacts with the γ-chain and *clfB* with the α- and β-chains—thereby contributing to immune evasion and the persistence of chronic infections [19,47,48]. Additionally, Protein A exhibits affinity for the Fc region of immunoglobulin G and von Willebrand factor, thereby further compromising host immune defenses [49]. Strains that express *bbp*, as well as collagen- and fibronectin-binding proteins, have been correlated with the pathogenesis of osteomyelitis [50].

*S. aureus* augments tissue invasiveness and immune evasion through the secretion of α-, β-, and γ-hemolysins, along with Panton–Valentine leukocidin, which collectively form pores in host cell membranes and instigate cellular lysis [51,52]. While α-hemolysin specifically targets platelets and monocytes, PVL—encoded by *lpv*—selectively affects leukocytes and has been implicated in necrotizing skin infections and methicillin-resistant *S. aureus* pneumonia [19,53,54]. Although γ-hemolysin, encoded by *hlg*, was found to be expressed in less than 20% of our isolates, it is noteworthy that all strains demonstrated some degree of hemolytic activity [19,55].

Coagulase plays a significant role in immune evasion by forming staphylothrombin complexes with prothrombin, which facilitates localized fibrin clot formation [19]. Lecithinase and lipase contribute to the degradation of phosphatidylcholine, thereby enhancing tissue invasion, while hemolysins promote iron acquisition through the breakdown of hemoglobin [56,57,58]. Esculinase catalyzes the hydrolysis of esculin, resulting in the production of esculetol that chelates iron, a critical factor for the expression of virulence determinants [56,57]. Caseinase and gelatinase compromise the integrity of connective tissue, thereby enhancing the organism’s invasiveness, while DNase and amylase facilitate immune evasion and metabolic processes [56,57,58,59].

## 4. Materials and Methods

### 4.1. Patient Selection and Sample Processing

Fifty-five adult oncology patients who developed EGFR inhibitor–associated CARs were enrolled in our study. *S. aureus* identification was based on colony morphology, Gram staining, catalase and coagulase positivity, and confirmation via the VITEK^®^ 2 Compact system (bioMérieux, Marcy-l’Étoile, France).

Only those with microbiologically confirmed *S. aureus* colonization or infection were selected for microbiological and molecular analysis. A total of 42 *S. aureus* strains were isolated from skin lesions and underwent phenotypic and genotypic characterization for antibiotic resistance and virulence.

### 4.2. Antibiotic Susceptibility Testing

Phenotypic antibiotic susceptibility was assessed using the Kirby–Bauer disk diffusion methodology on Mueller–Hinton agar in accordance with the established guidelines of the 2014 Clinical and Laboratory Standards Institute (CLSI) and subsequently verified through the automated VITEK 2 Compact system [60,61,62]. The resistance profiles were classified within the framework of multidrug-resistant (MDR), extensively drug-resistant (XDR), and pandrug-resistant (PDR) bacteria, as delineated by Magiorakos et al. (2012) [63].

Nevertheless, it is important to note that the classification was contingent upon the spectrum of antibiotics encompassed within the local testing panel available at the time of the investigation. Not all antimicrobial categories listed by Magiorakos et al. (2012) [63] were subjected to testing; consequently, the MDR/XDR/PDR classification was employed within a constrained context and should be interpreted accordingly. This limitation has been acknowledged, and the terminology used is primarily intended to facilitate a comparative discourse regarding resistance patterns.

### 4.3. Biofilm Formation Assay

The biofilm-producing ability of *S. aureus* isolates was evaluated using the standard microtiter plate assay. The isolates were cultivated in Tryptic Soy Broth (TSB) without glucose supplementation, adjusted to a turbidity equivalent to 0.5 McFarland standard (approximately 1.5 × 10^8^ CFU/mL). A volume of 10 µL of this standardized inoculum was added to each well of a 96-well microtiter plate containing fresh TSB and incubated at 37 °C for 24, 48, and 72 h [64,65]. After incubation, wells were gently washed, stained with 0.1% crystal violet, and the dye retained by the adherent biofilm was solubilized using 33% acetic acid. The optical density was measured at 492 nm to quantify biofilm biomass [22,65].

### 4.4. Phenotypic Detection of Soluble Virulence Factors

Phenotypic identification of soluble virulence determinants in *S. aureus* was conducted through spot inoculation on nutrient agar media augmented with enzyme-specific substrates. The enzymatic virulence determinants subjected to evaluation included: hemolysins (α and β), amylase, caseinase, gelatinase, esculinase, DNase, lipase, and lecithinase. Reactions were interpreted based on the appearance of characteristic halos or precipitates as described in previous literature [19,54,60].

Hemolysins were examined on 7% sheep blood agar, incubated at 37 °C for a duration of 24 h. Hemolysis was categorized into β-hemolysis (complete, transparent halo), α-hemolysis (partial, greenish halo), or γ-hemolysis (absence of hemolysis). In certain instances, plates were stored at 4 °C for a period of 30 min prior to analysis to augment visualization. 

Lecithinase activity was evaluated on agar enriched with 2.5% egg yolk and incubated at 37 °C for a maximum of 7 days. A positive response was signified by an opaque zone (precipitation) resulting from diglyceride formation or a transparent halo due to lipid complex hydrolysis. 

Lipase was assessed utilizing agar fortified with 1% sorbitol monooleate and incubated at 37 °C for a maximum of 7 days. The presence of a white, opaque precipitation zone signaled enzymatic degradation of monooleate and the subsequent formation of insoluble calcium oleate crystals. 

DNase activity was identified on DNase agar augmented with methyl green, incubated at 37 °C for 24 h. The presence of a clear zone surrounding the colony indicated DNA hydrolysis. 

Esculinase activity was evaluated using agar composed of 1% esculin and 1% ferric ammonium citrate. Following 24 h of incubation at 37 °C, the darkening of the medium indicated the production of esculetol and subsequent chelation of iron. 

Caseinase activity was examined on agar containing 15% casein and incubated at 37 °C for 24 h. The manifestation of a precipitation zone indicated proteolytic breakdown of casein and the formation of insoluble paracaseinate. 

Gelatinase was assessed on agar containing 3% gelatin. Plates were incubated at 37 °C for 24 h, and the emergence of a clear halo post-refrigeration confirmed enzymatic activity. 

Amylase was evaluated on starch agar incubated at 37 °C for 24 h, followed by staining with Lugol’s iodine. A yellow halo surrounding colonies on a blue background indicated the hydrolysis of starch. 

Each strain was evaluated in duplicate. Only consistently positive results were documented, and all assays were interpreted visually by trained personnel utilizing standardized criteria.

### 4.5. Genotypic Characterization of Virulence and Resistance Markers

Genomic DNA was extracted using an alkaline lysis method, and its concentration and purity were verified by agarose gel electrophoresis [19]. Virulence genes—*bbp*, *clfA*, *clfB*, *ebps*, *fnbB*, *fib*, *lpv*, and *hlg*—were detected using multiplex polymerase chain reaction (PCR) based on the protocol by Cotar et al. [19] (Table 10, Table 11 and Table 12, Supplementary Table S2). enotypic methicillin resistance profiling was performed through multiplex PCR targeting *S. aureus* SCC*mec* cassette types I–V, using primer sets and amplification protocols described by Milheiriço et al. and Zhang et al. [66,67] (Table 10, Table 11 and Table 12). PCR amplification was performed with GoTaq^®^ Green Master Mix (Jena Bioscience, Jena, Germany), and reactions were carried out on a Corbett Thermal Cycler with specific cycling conditions for each gene group.

PCR amplicons were visualized by 1.5% agarose gel electrophoresis stained with ethidium bromide (10 µg/mL) under ultraviolet (UV) light and compared with 100 bp molecular weight markers (M-Bench Top 100 bp DNA Ladder, Promega, Madison, WI, USA).

### 4.6. Clinical Correlation

Clinical severity of skin reactions was graded using the common terminology criteria for adverse events CTCAE v4.03 scale and correlated with bacterial genotypic profiles, biofilm-forming ability, and expression of soluble virulence factors.

### 4.7. Statistical Analysis

Patient data were entered into an OpenOffice Calc database (version 4.1.1, Apache Software Foundation). Statistical analyses were conducted using SAS University Edition (SAS Institute Inc., Cary, NC, USA) and R version 3.3.0 (R Foundation for Statistical Computing, Vienna, Austria), with supplementary analyses performed in RStudio version 0.99.902. Several R packages were used, including asbio, boot, bootstrap, and arm.

Descriptive statistics included the calculation of absolute and relative frequencies for categorical variables. For continuous variables, central tendency and variability were assessed using the mean, median, standard deviation, variance, and interquartile range (IQR). Distribution characteristics such as skewness and kurtosis were also computed. The Shapiro–Wilk test was applied to evaluate normality, and graphical representations included histograms, density plots, normal probability plots, and boxplots.

Inferential analyses were selected based on the distribution of the data. For variables approximating a normal distribution, Welch’s two-sample *t*-test was used to compare groups. For variables deviating from Gaussian distribution, a bootstrap resampling approach was employed to assess differences in medians. Comparisons involving more than two patient groups were conducted using analysis of variance (ANOVA). Associations between continuous variables were examined using Spearman’s rank correlation coefficient (ρ). A *p*-value below 0.05 was considered statistically significant.

## 5. Conclusions

*S. aureus* was identified in 90% of papulopustular and paronychia lesions associated with EGFR inhibitor therapy, with numerous strains demonstrating multidrug resistance (MDR)—particularly to β-lactams and tetracyclines—and a pronounced capacity for biofilm formation, especially in lesions exhibiting chronic progression. Virulence profiling revealed the frequent expression of pore-forming enzymes, esculinase, and caseinase, while DNase and esculinase expression showed a significant correlation with clinical severity.

From a genotypic standpoint, adhesion-related virulence genes (*clfA*, *clfB*, *fib*) were frequently expressed, whereas genes encoding exotoxins (*hlg*, *lpv*) were less commonly detected, suggesting a virulence profile that favors colonization and persistence over acute toxicity.

Notably, approximately one-third of patients harbored nasal strains that were both genetically and phenotypically identical to those isolated from skin lesions, suggesting that nasal colonization may serve as a reservoir for autoinoculation and recurrence. This finding underscores the need for routine nasal screening and decolonization strategies in patients initiating EGFR inhibitor therapy, particularly those with recurrent or severe cutaneous adverse reactions (CARs).

The enzymatic score—especially when based on DNase and esculinase expression levels—showed strong predictive value for CAR severity and may serve as a promising clinical biomarker. Its simplicity and accessibility support further validation in prospective clinical trials.

Collectively, these findings highlight the pivotal role of *S. aureus* in the pathogenesis of CARs and support the integration of routine microbial sampling and antibiogram-guided therapy into standard management. Future research should explore molecular distinctions between early- and late-onset eruptions and validate enzymatic scoring as a tool for risk stratification and personalized therapeutic interventions.

## Figures and Tables

**Table 1:** Spearman correlation—biofilm vs. CTCAE 4.03 severity.

Spearman Correlation	CTCAE 4.03 Severity
Biofilm 24 h ρ Spearman (*p*-value)	0.0910 (0.5665)
Biofilm 48 h ρ Spearman (*p*-value)	0.1849 (0.2409)
Biofilm 72 h ρ Spearman (*p*-value)	0.1783 (0.2584)

**Table 2:** Spearman correlation—virulence enzymes vs. Common Terminology Criteria for Adverse Events (CTCAE v4.0) severity.

Virulence Factors	CTCAE 4.0 Severity
Esculinase ρ Spearman (*p*-value)	0.23954 (0.1265)
DNase ρ Spearman (*p*-value)	0.30797 (0.04723)
Lipase ρ Spearman (*p*-value)	0.0979 (0.5374)
Caseinase ρ Spearman (*p*-value)	−0.01885 (0.9057)
Gelatinase ρ Spearman (*p*-value)	0.1430 (0.3663)
Lecithinase ρ Spearman (*p*-value)	0.10351 (0.5142)
Hemolysin ρ Spearman (*p*-value)	0.09876 (0.5338)

**Table 3:** Principal component analysis—virulence markers.

Principal Component (PC)	Explained Variance Proportion	Cumulative Explained Variance
PC1	0.2769	0.2769
PC2	0.2550	0.5319
PC3	0.1566	0.6885
PC4	0.1256	0.8142
PC5	0.0859	0.9001
PC6	0.0618	0.9620
PC7	0.0379	1.0000

**Table 4:** Enzymatic score distribution.

Enzymatic Score	Absolute Frequency (Relative Frequency %)
0	9/42 (21.42)
1	14/42 (33.33)
2	14/42 (33.33)
3	5/42 (11.90)

**Table 5:** Multinomial logistic regression—enzymatic score and Common Terminology Criteria for Adverse Events (CTCAE v4.0) severity.

Variable	β	S.E.	*p*-Value	Exp(β) (Odds Ratio)	95% CI for Exp(β)
Intercept 1	−0.8536	0.3992	0.9625	-	-
Intercept 2	2.0487	0.4934	<0.0001	-	-
Enzymatic Score 0	REFERENCE	-	-	-	-
Enzymatic Score 1	−0.4125	0.5171	0.4251	2.051	0.40 to 10.36
Enzymatic Score 2	−0.2335	0.5700	0.6515	2.453	0.48 to 12.50
Enzymatic Score 3	1.7767	0.8878	0.0454	18.310	1.37 to 244.15

**Table 6:** Frequency of adhesion and toxin genes in *S. aureus* strains.

Gene	Absolute/Relative Frequency
*hlg*	7/42 (16.66)
*fib*	15/42 (35.71)
*clfA*	36/42 (85.71)
*clfB*	36/42 (85.71)
*bbp*	6/42 (14.28)

**Table 7:** Frequency of antibiotic resistance in *S. aureus* strains.

Antibiotic Resistance	Absolute/Relative Frequency
Penicillin	35/42 (83.33)
Methicilin (Cefoxitin)	7/42 (16.66)
Erythromycin	19/42 (45.23)
Clindamycin	18/42 (42.85)
Biseptol	2/42 (4.76)
Tetracycline	24/42 (57.14)
Doxycycline	24/42 (57.14)
Kanamycin	5/42 (11.90)

**Table 8:** CTCAE severity scores in patients with MRSA infected cutaneous adverse reactions.

Isolate ID	CTCAE Severity Score
a41	Grade 3
a8	Grade 1
a2	Grade 3
a18	Grade 3
a16	Grade 3
a15	Grade 1
a9	Grade 3

**Table 9:** Frequency of SCC*mec* and resistance genes in *S. aureus* strains.

Gene	Absolute/Relative Frequency
type I	2/42 (4.76)
type IV a	2/42 (4.76)
type IV c	2/42 (4.76)
type IV d	5/42 (11.90)
*kdp*	1/42 (2.38)
*mec A*	3/42 (7.14)
*mec I*	4/42 (9.52)
*ccr B2*	3/42 (7.14)
SCC*mec* III J1	2/42 (4.76)

**Table 10:** Primer sequences, amplicon sizes, and specificities used for the detection of virulence and antibiotic resistance genes in *S. aureus* isolates based on the protocols of Cotar et al. (2010), Milheiriço et al. (2007), and Zhang et al. (2005) [19,66,67].

Target Gene/SCCmec Type	Amplicon Size (bp)	Primers	Nucleotide Sequences	Target Specificity/SCCmec Region
*bbp*	575	BBP-1/BBP-2	5′–AACTACATCTAGTACTCAACAACAG–3′/5′–ATGTGCTTGAATAACACACTACTCT–3′	Virulence
*ebpS*	186	EBP-1/EBP-2	5′–CATCCAGAACCAATCGAAGAC–3′/5′–CTTAACAGTTACATCATCATGTTTTACTTTG–3′	Virulence
*clfA*	292	CLFA-1/CLFA-2	5′–ATTGGCGTGGCTTCAGTGCT–3′/5′–CGTTTCTTCCGTAGTTGCATTTG–3′	Virulence
*clfB*	205	CLFB-1/CLFB-2	5′–ACATCAGTAATAGTAGGGGGCAAC–3′/5′–TTCGCACTGTTTGTGTTTGCAC–3′	Virulence
*fnbB*	524	FNBB-1/FNBB-2	5′–GTAACAGCTAATGGTGCGAATTGATACT–3′/5′–CAAGTTCGATAGGAGTACTAGTTTC–3′	Virulence
*fib*	404	FIB-1/FIB-2	5′–CTACAACTACAATTGCCGTCAACAG–3′/5′–GCTCTTGTAAAGCCATTTTCTTCAC–3′	Virulence
*lpv*	443	luk-PV-1/luk-PV-2	5′–ATCATTAGGTAAAATGTCTGGACATGATCCA–3′/5′–GCATCAASTGTATTGGATAGCAAAAGC–3′	Virulence
*hlg*	937	HLG-1/HLG-2	5′–GCCAATCCGTTATTAGAAATGC–3′/5′–CCATAGACGTAGCAACGGAT–3′	Virulence
*SCCmec I* (*J1*)	495	CIF2 F2/CIF2 R2	5′–TTC GAG TTG CTG ATG AAG AAG G–3′/5′–ATT TAC CAC AAG GAC TAC CAG C–3′	SCCmec I, region J1
*SCCmec III* (*J3*)	414	RIF5 F10/RIF5 R13	5′–TTC TTA AGT ACA CGC TGA ATC G–3′/5′–GTC ACA GTA ATT CCA TCA ATG C–3′	SCCmec III, region J3
*ccr complex*	311	ccrB2 F2/ccrB2 R2	5′–AGT TTC TCA GAA TTC GAA CG–3′/5′–CCG ATA TAG AAW GGG TTA GC–3′	SCCmec II & IV, ccr
*mec complex*	209	mecI P2/mecI P3	5′–ATC AAG ACT TGC ATT CAG GC–3′/5′–GCG GTT TCA ATT CAC TTG TC–3′	SCCmec II & III, mecI
*mecA gene*	162	mecA P4/mecA P7	5′–TCC AGA TTA CAA CTT CAC CAG G–3′/5′–CCA CTT CAT ATC TTG TAA CG–3′	mecA gene
*SCCmec V* (*J1*)	377	SCCmecV J1 F/SCCmecV J1 R	5′–TTC TCC ATT CTT GTT CAT CC–3′/5′–AGA GAC TAC TGA CTT AAG TGG–3′	SCCmec V, region J1
*Regions I–VI* (*J3*)	342	dcs F2/dcs R1	5′–CAT CCT ATG ATA GCT TGG TC–3′/5′–CTA AAT CAT AGC CAT GAC CG–3′	Regions I–VI, J3
*SCCmec II* (*J1*)	284	kdp F1/kdp R1	5′–AAT CAT CTG CCA TTG GTG ATG C–3′/5′–CGA ATG AAG TGA AAG AAA GTG G–3′	SCCmec II, region J1
*SCCmec III* (*J1*)	243	SCCmec III J1 F/SCCmec III J1 R	5′–CAT TTG TGA AAC ACA GTA CG–3′/5′–GTT ATT GAG ACT CCT AAA GC–3′	SCCmec III, region J1
*SCCmec I*	613	Type I-F/Type I-R	5′–GCT TTA AAG AGT GTC GTT ACA GG–3′/5′–GTT CTC TCA TAG TAT GAC GTC C–3′	SCCmec I
*SCCmec II*	398	Type II-F/Type II-R	5′–CGT TGA AGA TGA TGA AGC G–3′/5′–CGA AAT CAA TGG TTA ATG GAC C–3′	SCCmec II
*SCCmec III*	280	Type III-F/Type III-R	5′–CCA TAT TGT GTA CGA TGC G–3′/5′–CCT TAG TTG TCG TAA CAG ATC G–3′	SCCmec III
*SCCmec IVa*	776	Type IVa-F/Type IVa-R	5′–GCC TTA TTC GAA GAA ACC G–3′/5′–CTA CTC TTC TGA AAA GCG TCG–3′	SCCmec IVa
*SCCmec IVb*	493	Type IVb-F/Type IVb-R	5′–TCT GGA ATT ACT TCA GCT GC–3′/5′–AAA CAA TAT TGC TCT CCC TC–3′	SCCmec IVb
*SCCmec IVc*	200	Type IVc1-F/Type IVc1-R	5′–TCT ATT CAA TCG TTC TCG TAT T–3′/5′–TCG TTG TCA TTT AAT TCT GAA CT–3′	SCCmec IVc
*SCCmec IVd*	881	Type IVd1-F/Type IVd1-R	5′–AAT TCA CCC GTA CCT GAG AA–3′/5′–AGA ATG TGG TTA TAA GAT AGC TA–3′	SCCmec IVd
*SCCmec V*	325	Type V-F/Type V-R	5′–GAA CAT TGT TAC TTA AAT GAG CG–3′/5′–TGA AAG TTG TAC CCT TGA CAC C–3′	SCCmec V
*ccr Type 5*	495	ccrC-F/ccrC-R	5′–CGT CTA TTA CAA GAT GTT AAG GAT AAT–3′/5′–CCT TTA TAG ACT GGA TTA TTC AAA ATA T–3′	ccr Type 5

**Table 11:** PCR conditions used for amplification of virulence genes in isolated *S. aureus* strains, according to Cotar et al. (2010) [19].

Gene	Initial Denaturation	Number of Cycles	Denaturation Per Cycle	Primer Annealing	Primer Extension	Final Extension
*Lpv*	94 °C, 5 min	30	94 °C, 30 s	55 °C, 30 s	72 °C, 1 min	72 °C, 10 min
*hlg*	94 °C, 5 min	30	94 °C, 30 s	55 °C, 30 s	72 °C, 1 min	72 °C, 10 min
*bbp*	94 °C, 5 min	30	94 °C, 30 s	55 °C, 30 s	72 °C, 1 min	72 °C, 10 min
*ebpS*	94 °C, 5 min	30	94 °C, 30 s	55 °C, 30 s	72 °C, 1 min	72 °C, 10 min
*clfA*	94 °C, 5 min	25	94 °C, 1 min	55 °C, 1 min	72 °C, 1 min	72 °C, 10 min
*clfB*	94 °C, 5 min	25	94 °C, 1 min	55 °C, 1 min	72 °C, 1 min	72 °C, 10 min
*fnbB*	94 °C, 5 min	25	94 °C, 1 min	55 °C, 1 min	72 °C, 1 min	72 °C, 10 min
*fib*	94 °C, 5 min	25	94 °C, 1 min	55 °C, 1 min	72 °C, 1 min	72 °C, 10 min

**Table 12:** PCR reaction volumes of different reagents (after Milheiriço et al., 2007) [66].

Primer Volume	Master Mix PCR Volume DreamTaq Green (Thermo Scientific, SUA, Waltham, MA, USA)	Ultrapure Water Volume	DNA Volume	Total Reaction Volume
0.3 µL	10 µL	6.5 µL	0.5 µL	20 µL

## Data Availability

Data are contained within the article and Supplementary Materials.

## Supplementary Materials

The following supporting information can be downloaded at: https://www.mdpi.com/article/10.3390/ijms26146595/s1.

## Abbreviations

The following abbreviations are used in this manuscript:

CTCAE	Common Terminology Criteria for Adverse Events
EGFR	Epidermal Growth Factor Receptor
95% CI	95% Confidence Interval (calculated using the Student’s t distribution)
IQR	Interquartile Range (difference between the 75th and 25th percentiles)
MDR	Multidrug-Resistant
PDR	Pandrug-Resistant
PCR	Polymerase Chain Reaction
*S. aureus*	*Staphylococcus aureus*
XDR	Extensively Drug-Resistant
